# Internet-Delivered Cognitive Behavioral Therapy Tailored to Spouses and Significant Others of Public Safety Personnel: Formative Evaluation Study

**DOI:** 10.2196/51088

**Published:** 2023-09-27

**Authors:** Heather D Hadjistavropoulos, Sarah J Reiser, Janine D Beahm, Hugh C McCall, Isabelle Dena, Abby R Phillips, Melissa Scheltgen, Shimona Sekhar, Marilyn Cox, Heidi Cramm, Nathalie Reid

**Affiliations:** 1 Department of Psychology University of Regina Regina, SK Canada; 2 PSPNET University of Regina Regina, SK Canada; 3 Department of Family Studies & Gerontology Mount Saint Vincent University Halifax, NS Canada; 4 School of Rehabilitation Therapy Queen's University Kingston, ON Canada; 5 Child Trauma Research Centre University of Regina Regina, SK Canada

**Keywords:** internet-delivered cognitive behavioral therapy, ICBT, internet interventions, transdiagnostic, spouses and significant others, public safety personnel, formative evaluation

## Abstract

**Background:**

Spouses and significant others (SSOs) of public safety personnel (PSP) are affected by the risks and requirements of these occupations. Internet-delivered cognitive behavioral therapy (ICBT) provides a convenient and accessible treatment format that can be tailored to the needs of SSOs of PSP.

**Objective:**

This study aimed to assess the initial use and client perceptions (eg, likes, helpfulness, and areas for improvement) of a self-guided, transdiagnostic ICBT course designed for Canadian SSOs of PSP and identify opportunities to further tailor ICBT for this group.

**Methods:**

SSOs were invited to complete a 5-lesson, self-guided, transdiagnostic ICBT course. Descriptive statistics were used to analyze the demographic and clinical characteristics of participants. Content analysis was used to analyze the data from open-ended survey responses and interviews to understand their experiences with ICBT.

**Results:**

Clients (N=118) endorsed various mental health concerns (eg, depression, anxiety, posttraumatic stress symptoms, and relationship concerns) with a range of severity levels. Most clients identified as White (110/116, 94.8%) and women (108/116, 91.5%), with a mean age of 42.03 (SD 9.36) years. Of the 26 clients who were interviewed, 89% (23/26) reported believing that ICBT is helpful and 92% (24/26) reported finding at least 1 skill helpful. Clients provided suggestions for course improvements. On the basis of this feedback and quantitative data, changes were made to areas such as the delivery of materials, content, case stories, and timelines. Overall, the results indicated that many SSOs of PSP had positive perceptions of ICBT tailored to their needs and found several aspects of the course helpful, supporting the continued delivery of tailored ICBT to this population. However, there remains a need for continued promotion of the course and outreach to diverse groups of SSOs of PSP.

**Conclusions:**

Findings from this formative evaluation provide insight into the unique experiences and needs of SSOs of PSP and provide preliminary evidence for the use of tailored ICBT to support the mental health of this group in Canada.

## Introduction

### Background

Public safety personnel (PSP) include border services officers, public safety communicators, correctional workers, firefighters (career and volunteer), Indigenous emergency managers, operational intelligence personnel, paramedics, police, search and rescue personnel, and those in other occupations that uphold the safety and security of communities [1]. PSP encounter significant occupational stressors [2] and an increased risk of experiencing symptoms of mental health disorders [3], all of which can affect family relationships. Spouses and significant others (SSOs) of PSP are impacted by the demands of public safety occupations [4]. SSOs of PSP report chronic and cumulative stressors related to both the risks and requirements of public safety work and their provision of emotional and instrumental support for their PSP significant other [5]. The demands of PSP work (eg, shiftwork, dangerous environments, and trauma exposure) impact the couple relationship and family functioning [6-8]. SSOs of PSP may experience worry regarding the risks of PSP work, indirect trauma, and other negative mental health consequences [4,9,10]. Mental health resources and supports for both PSP and their SSOs are essential to support the well-being and resilience of PSP families [11].

Recognizing the need for mental health supports designed for PSP, the Government of Canada’s National Action Plan for Addressing Post Traumatic Stress Injuries identified internet-delivered cognitive behavioral therapy (ICBT) as a valuable tool for providing mental health care to Canadian PSP [12]. This action plan led to the development of PSPNET, which delivers ICBT tailored to the needs of Canadian PSP. ICBT provides cognitive behavioral treatment materials through a web-based course, a format that has garnered extensive research support for the treatment of posttraumatic stress disorder (PTSD), depression, and anxiety disorders [13-15]. Of note, ICBT clinics typically refer clients presenting with severe and complex mental health problems (eg, psychosis, high risk of suicide, and severe substance use disorders) to more appropriate services [16]. ICBT can be delivered in a scalable and cost-effective manner [17] and addresses several barriers to face-to-face therapy, including barriers related to transportation, concerns about privacy and stigma, time constraints, and treatment costs [18,19]. Self-guided ICBT (ie, ICBT without a therapist) is particularly scalable [20] and cost-effective [21] and has demonstrated good outcomes for treating anxiety, depression [22], and PTSD [15], although therapist-guided ICBT tends to be somewhat more effective [23,24]. Research has shown that clients of both therapist-guided [17] and self-guided ICBT [25-27] maintain treatment gains at 1 year or longer after treatment.

PSPNET’s ICBT courses were developed using interview and survey feedback from PSP stakeholders [28,29]. Initially, the PSPNET team adapted an existing transdiagnostic ICBT course that had demonstrated effectiveness in the general population in Australia and the Canadian province of Saskatchewan [30-33]. This course was tailored for Canadian PSP and was titled the *PSP Wellbeing Course.* Initial research results from the *PSP Wellbeing Course* showed that tailored ICBT was used by PSP to address both occupational and personal concerns [34] and was viewed as beneficial [35]. Clients of the *PSP Wellbeing Course* showed good engagement and reported high treatment satisfaction, with 77% completing all modules of the course and 98% reporting that the course was worth their time [36]. Clients who reported clinically significant difficulties with depression, anxiety, PTSD, panic, or anger before treatment reported large improvements in these symptoms after taking the course [36]. A recent study showed that the *PSP PTSD Course* (a disorder-specific ICBT program for PTSD tailored to PSP) showed outcomes comparable with those of the *PSP Wellbeing Course* [37].

The results of qualitative research with PSPNET clients also yielded important insights into the challenges faced by PSP families and couples [34] and demonstrated a need to extend ICBT to SSOs of PSP. Specifically, PSP reported the following themes related to family life: communication and anger issues, withdrawal or lack of intimacy, lack of work-life balance, inadequate support from spouse or partner, scheduling conflicts, and vigilance regarding family safety. PSP clients reported an interest in having more support related to relationship and family issues. In addition, SSOs have contacted PSPNET to express their interest in participating in ICBT, noting a lack of accessible support that recognizes their unique challenges. Therefore, we sought to create and assess the first ICBT program specifically designed to support SSOs of PSP. PSPNET’s expansion of ICBT services to this group based on its initial research findings is consistent with a learning health system model [38], whereby data are continually collected and analyzed to inform iterative changes to a service.

This study focused on the development, delivery, and formative evaluation of a self-guided, transdiagnostic ICBT course tailored to SSOs of PSP (the *SSO Wellbeing Course*). The *SSO Wellbeing Course* was adapted from the *PSP Wellbeing Course* offered through PSPNET and was part of a larger project (PSPNET Families) that focused on creating and disseminating web-based evidence-based resources and supports for family members of PSP. The *SSO Wellbeing Course* represents the first specialized ICBT program to support the mental health and well-being of SSOs of PSP across Canada. As such, the evaluation focused on assessing usability and acceptance, which is an integral first step in evaluating the effectiveness of new tailored interventions [39]. A mixed methods approach, including both quantitative and qualitative data, was used to identify opportunities to improve ICBT for this group.

### Objectives

The main objectives of this research were to (1) assess initial course use (eg, client demographic and clinical characteristics, progress through course, and use of additional resources and discussion forum), (2) identify clients’ perceptions of the course (eg, likes, helpfulness, and areas for improvement), and (3) use data to inform course improvements. To meet these objectives, data were collected from multiple sources, including interviews, open-response questionnaire items, observational data (eg, the number of lessons accessed by clients), and symptom and relationship measures. Owing to the exploratory nature of the research, no hypotheses were made.

## Methods

### Setting

PSPNET was launched in 2019 to provide ICBT to Canadian PSP. In 2022, contract funding was secured to expand PSPNET to create PSPNET Families, a web-based well-being hub that provides information and resources to support family members of PSP. The development, delivery, and evaluation of the *SSO Wellbeing Course* constitute 1 component of the PSPNET Families project.

### Ethical Considerations

This study was approved by the Research Ethics Board of the University of Regina (#2022-054). All client data were stored on a secure server and deidentified before analysis. Besides granting participants access to the *SSO Wellbeing Course*, we offered no incentives to encourage participation. All clients of the *SSO Wellbeing Course* were made aware of these and many other details of this study, and they voluntarily provided informed consent before participating.

### Course Development

Consistent with the *PSP Wellbeing Course*, the *SSO Wellbeing Course* included 5 web-based lessons that were released gradually over 8 weeks and included weekly automated emails and messages within the intervention designed to prompt lesson completion. The lessons addressed (1) symptom identification and the cognitive behavioral model, (2) thought monitoring and challenging, (3) managing physical symptoms with dearousal strategies and pleasant activity scheduling, (4) managing unhelpful behaviors and graded exposure, and (5) relapse prevention. Each lesson contained the following: a slide show, downloadable and printable readings and activities, frequently asked questions, illustrative fictional case stories of individuals who took the course, and additional resources on various topics (eg, PTSD, communication skills, and sleep) available for clients to access based on their needs and interests. Written lesson materials were designed using Microsoft PowerPoint. Clients accessed all course materials by logging into their accounts on PSPNET’s website.

The *SSO Wellbeing Course* was offered as a self-guided program. To support the safety of clients, PSPNET Families clinicians completed weekly safety checks (ie, review of symptom measure scores) to monitor elevated scores and followed up with clients by phone if potential safety concerns were identified. The development of the *SSO Wellbeing Course* was guided by user-centered design principles, which endorse the active involvement of target users in the design process from the outset and the importance of a user-centered approach together with empirical research [40]. Adapting the course to the experiences and needs of SSOs was informed by regular feedback and suggestions from a working group (WG) consisting of 5 SSOs from across Canada whose significant others worked in various public safety sectors (ie, police, fire, and corrections). WG members participated in 6 meetings over the course of the project and provided integral information about their experiences, preferences, and needs as SSOs that informed content adaptations, visual presentation of course materials, and ideas for outreach strategies. Once launched, WG members were given access to the course and had the option of participating and providing feedback on the user experience.

Adaptations made to the *PSP Wellbeing Course* to create the *SSO Wellbeing Course* included adding (1) information on the unique lifestyle dimensions of PSP families to lesson 1, (2) new overview videos for each lesson, (3) a therapist-moderated discussion forum as an optional component of the course to provide a platform for peer discussion and support, and (4) new case stories about SSOs. The case stories represented the primary adaptation to the course and were used throughout the course to provide relatable examples of symptoms, challenges, and skill application. The story characters were based on the clinical team’s knowledge and the experiences shared by WG members. WG information helped to create and refine the stories, and WG members provided specific feedback on the SSO stories regarding content, relatability, diversity, helpfulness, visual presentation, and character photos. The course initially included stories of 7 fictional SSO characters with diverse backgrounds in terms of age, vocation, ethnicity, gender identity, family structure, and the PSP occupation in which their spouses or significant others worked. Each lesson included stories (eg, reflections and homework examples) from several characters. Consistent with the Shaffer and Zikmund-Fisher [41] taxonomy, the purpose of the stories was to enhance learning, increase engagement, model the implementation of skills and helpful behaviors, persuade clients to practice skills, and comfort and validate them. Stories have been found to help improve client understanding, enhance support, and potentially impact behavioral change [42].

### Eligibility and Recruitment

The *SSO Wellbeing Course* was designed to be implemented and evaluated across Canada. Prospective clients were deemed eligible to participate if they self-reported that they were (1) a current or former SSO of a current or former PSP; (2) aged ≥18 years; (3) a Canadian resident; (4) able to regularly access the internet through a computer; and (5) not reporting a primary problem of psychosis, mania, or substance use.

Recruitment began in July 2022 and is ongoing. Data collected as recently as June 2023 from clients who enrolled as recently as May 2023 were used in this evaluation. The course was advertised through Google Ads, the PSPNET website, presentations and attending conferences, PSPNET’s social media pages, the use of social media influencers (ie, an SSO and a PSP), and direct contact with PSP organizations and other groups to request that they inform their members about the course (eg, via email). Interested SSOs were directed to a website with information about the course and instructions for enrollment. Following the provision of informed consent, clients were asked to complete screening questionnaires assessing their demographic characteristics, symptoms of mental health disorders, and relationship satisfaction and functioning. Subsequently, clients received secure log-in information and could begin the course. Clients who participated in the interviews also provided verbal consent by phone before beginning the interview process.

### Measures and Data Sources

#### Demographics Questionnaire

Sociodemographic information was collected as part of the web-based screening before the participants accessed the course materials. The demographics questionnaire queried information such as self-identified gender, race and ethnicity, age, community size, and the PSP sector of SSOs’ partners.

#### Clinical Measures

Baseline scores for mental health symptoms were assessed as part of the web-based screening and included the Patient Health Questionnaire-9 (PHQ-9) for depression [43], the Generalized Anxiety Disorder-7 (GAD-7) for anxiety [44], and the PTSD Checklist for DSM-5 for posttraumatic stress (PCL-5) [45]. Relationship satisfaction was assessed using the 4-item version of the Couples Satisfaction Index [46], and relationship functioning was assessed using the General Functioning subscale of the McMaster Family Assessment Device [47], which was adapted for this study (ie, minor changes to the language) to focus specifically on the couple relationship. These 5 measures have all been shown to be reliable and valid in previous research [43-47].

#### Semistructured Interviews

We invited 71 clients for interviews and conducted 26 semistructured interviews until we reached a point of saturation in identifying recurring themes. After 6 weeks of enrollment, regardless of how much of the course SSOs had completed, clients were sent an email inviting them to take part in a follow-up interview designed to gain feedback on their experiences with the course. The semistructured interview guide included questions about clients’ experiences with the course, including overall impacts and what they found helpful, unhelpful, or challenging, as well as clients’ preferences, including what they liked or wanted improved. All interviews were conducted by ID, who is a PSPNET clinical research associate with a master’s degree in social work. Interviews took place over the phone between September 1, 2022, and January 31, 2023, lasting between 15 and 30 minutes. All interviews were recorded and transcribed by a professional transcription service.

#### Open-Ended Questionnaire Items

Client feedback was collected using two types of open-ended questionnaires: (1) a biweekly course use questionnaire and (2) a treatment satisfaction questionnaire (TSQ). The course use questionnaire was administered at 2, 4, 6, and 8 weeks after enrollment and included 2 open-ended questions about aspects of the case stories and the *SSO Wellbeing Course* overall that clients wanted improved. The TSQ was a bespoke measure administered at 8 weeks after enrollment and included 4 open-ended questions asking what clients liked and wanted improved regarding case stories and overall course.

### Analysis

We used a mixed methods approach. Descriptive statistics were used to summarize the quantitative data (ie, demographic and clinical characteristics and course use data). Qualitative content analysis [48] was used to analyze qualitative data (ie, open-ended questionnaires and interview data). Quantitative data were used to address objective 1 (assessment of course use), qualitative data were used to address objective 2 (client perceptions of the course), and both sets of data were used to address objective 3 (inform course improvements). Data analyses were consistent with a learning health system model [38], which focuses on using data to inform improvements and further tailoring of course processes and materials to meet the specific needs of the users.

Conventional qualitative content analysis [48] was used to analyze all qualitative data, including the semistructured interviews and the open-ended questionnaire items. Conventional qualitative content analysis uses an inductive approach without preconceived categories [48]. Data from all open-ended questionnaire items were combined in the analysis, and interview data were analyzed separately from questionnaire data. All data were uploaded into the qualitative analysis software NVivo (Lumivero) [49]. Data were coded by meaning units into overarching categories and subcategories. The initial coding was conducted by JDB and then reviewed by ID. All disagreements were resolved through discussion.

## Results

### Quantitative Results: Client Characteristics and Course Use

**Figure 1:**
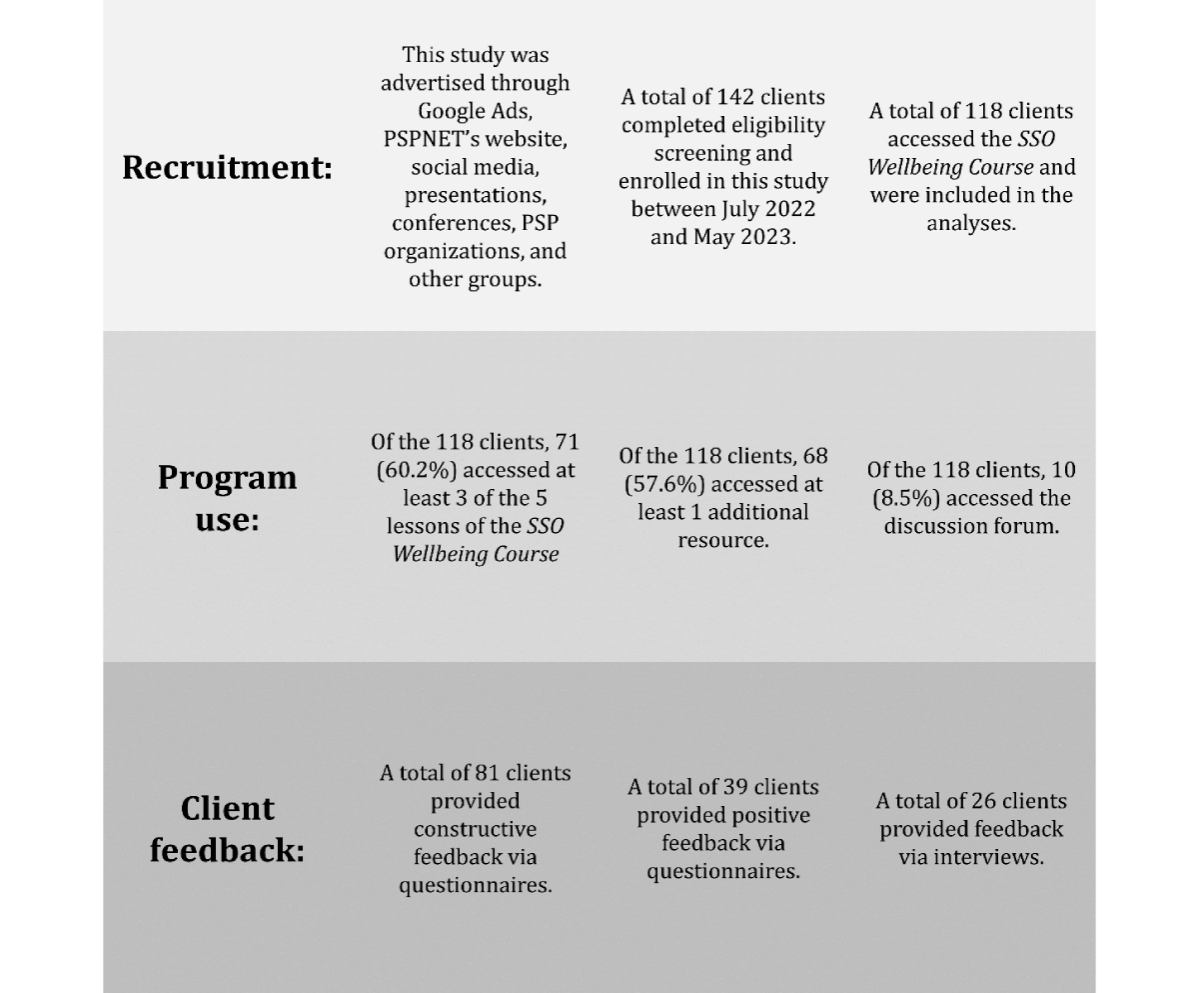
Overview of recruitment, program use, and client feedback. PSP: public safety personnel; SSO: spouse or significant other.

At the time of data analysis, 142 Canadian SSOs completed the web-based screening and were enrolled in the course, and 118 started the course. These 118 clients were aged between 22 and 71 years, with a mean age of 42.03 (SD 9.36) years. Most clients resided in the provinces of Ontario (36/116, 31%), Saskatchewan (23/116, 19.8%), or Alberta (17/116, 14.7%). Most clients identified as White (110/116, 94.8%), women (108/116, 93.1%), employed (97/116, 83.6%), and living in an area with a population of <100,000 (72/116, 62.1%). Most clients indicated that their significant other is or was employed in the police sector (61/116, 52.6%) and that they were currently, rather than formerly, married to or in a relationship with a PSP (114/116, 98.3%). Nearly half of the sample (53/116, 45.7%) had elevated scores in at least 1 symptom area. PSPNET Families clinicians conducted safety check phone calls with a total of 5 clients, owing to a score increase of ≥5 on any symptom measure or the endorsement of potential suicidal ideation (as measured by item 9 of the PHQ-9). Upon completion of a risk assessment, further follow-up was not required for these clients. Nearly half of the sample (43/101, 42.6%) reported relationship dissatisfaction on the 4-item version of the Couples Satisfaction Index, and most clients (66/116, 56.9%) reported moderate relationship functioning on the General Functioning subscale of the McMaster Family Assessment Device. The subset of 26 clients who completed the interviews reported similar demographic (Table 1) and clinical and relationship characteristics (Table 2) to the overall sample. An overview of the flow of participants through the study, including recruitment, program use, and the provision of feedback via questionnaires and interviews, is displayed in Figure 1.

Of the 118 clients who started the course, 71 (60.2%) accessed most lessons (ie, at least up to lesson 3). The most commonly used additional resource was *Communication with a Significant Other* (40/118, 33.9%). Details regarding progress through the lessons and use of additional resources are shown in Table 3.

The discussion forum had minimal use. A total of 18 user comments from 10 clients were posted. Despite efforts to engage clients through changes to the placement of the forum within the course, the addition of a video showing how to use the forum, and prompts from the therapist moderating the forum, most clients did not use the forum.

**Table 1:** Clients’ demographic characteristics (N=118).

Characteristic		Total sample^a^	Interviewees (n=26)^a^
**Gender, n (%)**			
	Woman	108 (91.5)	24 (96)
	Man	7 (5.9)	1 (4)
	Nonbinary	1 (0.8)	0 (0)
**Community size, n** **(%)**			
	<100,000	72 (62.1)	17 (68)
	>100,000	44 (37.9)	8 (32)
**Relationship status with PSP^b^, n (%)**			
	Currently with PSP	114 (98.3)	25 (100)
	Formerly with PSP	2 (1.7)	0 (0)
**Self-identified race and ethnicity, n (%)**			
	White	110 (94.8)	24 (96)
	Ethnocultural minority group	6 (5.2)	1 (4)
**Roles, n (%)**			
	Employed	97 (83.6)	19 (76)
	Runs household	63 (54.3)	15 (60)
**Highest level of education, n (%)**			
	High school	10 (8.6)	2 (8)
	Some college or university	13 (11.2)	3 (12)
	College diploma or university degree	77 (66.4)	17 (68)
	Professional or graduate degree	16 (13.8)	3 (12)
**Age (years)**			
	Mean (SD)	42.03 (9.36)	45.99 (10.53)
	22-29, n (%)	11 (9.5)	0 (0)
	30-39, n (%)	41 (35.3)	9 (36)
	40-49, n (%)	46 (39.7)	9 (36)
	50-59, n (%)	13 (11.2)	4 (16)
	60-71, n (%)	5 (4.3)	3 (12)
**Residing province or territory, n (%)**			
	Ontario	36 (31)	5 (20)
	Saskatchewan	23 (19.8)	7 (28)
	Alberta	17 (14.7)	6 (24)
	British Columbia	15 (12.9)	2 (8)
	Nova Scotia	7 (6)	0 (0)
	New Brunswick	6 (5.2)	3 (12)
	Prince Edward Island	5 (4.3)	1 (4)
	Manitoba	3 (2.6)	1 (4)
	Newfoundland and Labrador	2 (1.7)	0 (0)
	Yukon	2 (1.7)	0 (0)
**Significant other’s PSP sector, n** **(%)**			
	Police	61 (52.6)	17 (68)
	Fire	25 (21.6)	5 (20)
	Paramedicine	13 (11.2)	1 (4)
	Corrections	11 (9.5)	1 (4)
	Dispatch or communications	3 (2.6)	0 (0)
	Other (eg, border services)	3 (2.6)	1 (4)

^a^Not all clients responded to all questions. Percentages shown were calculated based on the number of valid responses (generally, 116 responses for the total sample and 25 for interviewees).

^b^PSP: public safety personnel.

**Table 2:** Clients’ clinical and relationship characteristics (N=118).

			Total sample^a^		Interviewees (n=26)^a^
**Treatments in the past month, n (%)**					
	Used mental health medication	35 (29.7)		6 (24)	
	Seen health care or mental health care provider for mental health reasons	46 (39)		7 (28)	
**Pretreatment PHQ-9^b^**					
	Mean (SD)	7.66 (5.82)		6.32 (5.48)	
	Minimal symptoms (0-4), n (%)	42 (36.2)		10 (40)	
	Mild symptoms (5-9), n (%)	38 (24.1)		10 (40)	
	Clinically significant (10-27), n (%)	36 (31)		5 (20)	
**Pretreatment GAD-7^c^**					
	Mean (SD)	7.30 (5.44)		6.48 (5.58)	
	None-minimal (0-4), n (%)	44 (37.9)		10 (40)	
	Mild symptoms (5-9), n (%)	34 (29.3)		10 (40)	
	Clinically significant (10-21), n (%)	38 (32.8)		5 (20)	
**Pretreatment PCL-5^d^**					
	Mean (SD)	19.34 (16.67)		16.28 (14.86)	
	Not clinically significant (0-32), n (%)	93 (80.2)		22 (88)	
	Clinically significant (33-80), n (%)	23 (19.8)		3 (12)	
**Number of clinically significant scores across PHQ-9, GAD-7, and PCL-5, n (%)**					
	0	63 (54.3)		18 (72)	
	1	22 (19)		3 (12)	
	2	18 (15.5)		2 (8)	
	3	13 (11.2)		2 (8)	
**Pretreatment CSI-4^e^**					
	Mean (SD)	13.57 (4.71)		13.87 (4.68)	
	Relationship dissatisfaction (0-13), n (%)	43 (42.6)		9 (39.1)	
	Relationship satisfaction (14-21), n (%)	58 (57.4)		14 (60.9)	
**Pretreatment GF-12^f^**					
	Mean (SD)	2.12 (0.54)		2.01 (0.57)	
	Higher relationship functioning (1.00-1.99), n (%)	45 (38.8)		14 (56)	
	Moderate relationship functioning (2.00-2.99), n (%)	66 (56.9)		9 (36)	
	Lower relationship functioning (3.00-4.00), n (%)	5 (4.2)		2 (8)	

^a^Not all clients responded to all questions. Percentages shown were calculated based on the number of valid responses (generally, 116 responses for the total sample and 25 for interviewees).

^b^PHQ-9: Patient Health Questionnaire-9.

^c^GAD-7: Generalized Anxiety Disorder-7.

^d^PCL-5: PTSD Checklist for DSM-5.

^e^CSI-4: 4-item version of the Couples Satisfaction Index.

^f^GF-12: General Functioning subscale of the McMaster Family Assessment Device.

**Table 3:** Program use (N=118).

			Total sample		Interviewees (n=26)
**Lessons accessed, n (%)**					
	Lesson 1	118 (100)		26 (100)	
	Lesson 2	89 (75.4)		24 (92.3)	
	Lesson 3	71 (60.2)		22 (84.6)	
	Lesson 4	60 (50.8)		20 (76.9)	
	Lesson 5	46 (39)		20 (76.9)	
**Top additional resources accessed, n (%)**					
	Communication with a significant other	40 (33.9)		12 (46.2)	
	PTSD^a^	34 (28.8)		15 (57.7)	
	Sleep	34 (28.8)		12 (46.2)	
	Building motivation	33 (28)		11 (42.3)	
	Anger	31 (26.3)		11 (42.3)	
	Problem-solving and worry	30 (25.4)		10 (38.5)	
	Alcohol use	28 (23.7)		10 (38.5)	
	Assertiveness	28 (23.7)		11 (42.3)	
	Managing beliefs	27 (22.9)		9 (34.6)	
	Communication skills	26 (22)		9 (34.6)	
	Panic	26 (22)		9 (34.6)	
	Health anxiety	25 (21.2)		11 (42.3)	
	Grief	24 (20.3)		8 (30.8)	
	Additional skills for changing maladaptive thoughts	23 (19.5)		9 (34.6)	
	Suicidal ideation	19 (16.1)		7 (26.9)	
	Pain	17 (14.4)		6 (23.1)	

^a^PTSD: posttraumatic stress disorder.

### Qualitative Results: Feedback on the Course

#### Interview Findings

Of the 26 clients who participated in the follow-up interviews for the *SSO Wellbeing Course*, 23 (88%) reported believing that ICBT is helpful, 2 (8%) reported believing that ICBT works in principle but could be improved through streamlining or timeline changes to create more successes with the course, and 1 (4%) reported that they did not progress far enough in the *SSO Wellbeing Course* to determine whether ICBT works because the course did not meet their specific needs. The reasons clients reported finding ICBT helpful or unhelpful are listed in Table 4.

Of the 26 interviewees, 24 (92%) reported finding at least 1 of the skills from the course helpful. A summary of the skills that clients found the most helpful is outlined in Table 5. Some clients endorsed >1 skill as the most helpful. Of the 26 clients, 2 (8%) reported not being far enough along in the course to implement any skills. Some clients (5/26, 19%) reported finding at least 1 of the skills challenging to implement. A few of these clients described challenges in implementing specific skills because they were outside of their comfort zone. However, with continued practice, these clients noted that the skills became easier to implement. In addition, 1 client stated that procrastination contributed to the challenges in implementing the skills.

When asked about discussion forum use, 25 of the 26 interviewees (96.2%) reported that they did not use the discussion forum. They reported the following reasons for not using the forum: the forum was too difficult to find or navigate (8/25, 32%), they did not have enough time (7/25, 28%), they felt uncomfortable sharing or chose not to share (7/25, 28%), and there was too little activity from others (3/25, 12%).

**Table 4:** Client feedback on whether internet-delivered cognitive behavioral therapy (ICBT) works or is helpful based on semistructured interview data (n=26).

Category and subcategory			Example quote		Clients, n (%)
**Yes, ICBT works or is helpful**					23 (88)
	Accessible and convenient	“It’s better than having to go in person because it’s more flexible. And what I love about it, there are people working and spouses of people working in these kinds of careers that are in isolated places—I’ve been there. [Laughs] This is so great. I think it’s so needed. I mean, there has not been anything like this that I know of, until now. So, I think it has the potential to be something pretty impactful and pretty helpful to spouses of public safety personnel for sure.”		13 (50)	
	Makes a difference in SSOs^a^ lives by providing information and skills for enhancing well-being and normalizing mental health challenges	“I think, because I just needed a little bit more support in managing my symptoms, that the program was really great because it taught me some new tricks and ways to manage and address what’s causing my symptoms that I could easily do from home.”		10 (38)	
	Is needed and important for SSOs	“I almost wish that every spouse of a first responder had to take this. I think it, whether they have had any trauma just to even prepare them for this could happen to you.”		5 (19)	
	Provides a first step toward getting help	“I think it’s, I want to say it’s like a gateway course. It’s probably like a gateway help. Helps the scenario. Where you know, you got to start somewhere and physically go to see somebody might be too much for some individuals.”		4 (15)	
Yes, with improvements			“Yeah, yeah, no, I think it’s a, I think it’s a valuable tool, definitely a valuable tool. Just streamlined, maybe streamlined, I think you could probably streamline it in places.”		2 (8)
Unsure			“I’m not sure, I think I have to withhold my answer to that, because I’m not progressing through it as fast as I was hoping and I don’t know if it’s because it’s just really not, it’s not meeting my challenges, I guess. But nor does face-to-face counselling.”		1 (4)

^a^SSO: spouse and significant other.

**Table 5:** Client feedback on helpful course skills based on semistructured interview data (n=26).

Category	Example quote	Clients, n (%)
Thought challenging	“The thought challenging section was probably, oh, I would dare say lifechanging for me, especially the timing of it and what was going on in our life. We were moving during this whole situation, and yeah, it was just so helpful to me.”	12 (46)
Controlled breathing	“The controlled breathing helped me be less reactive in my relationship with my husband and think through an answer. I tend to be not very assertive. I tend to be, one way or the other more aggressive or the other direction kind of more timid. So, you know, going through the breathing exercises helped me think through an appropriate answer.”	8 (31)
Recognizing symptoms and symptom cycles	“So far, I find the identification of the behaviors in the cycle to be the most interesting. So, while I knew that I had some behaviors where I was responding to anxiety or depression, there were others on the lists that I didn’t realise in fact were behaviors that were resulting from sort of an anxious response, so that was very helpful to me.”	6 (23)
Graded exposure	“I think the skill around when things feel overwhelming, and you know you can’t do these things. This could break it down into those sorts of micro-steps about tiny little chunks. Taking tiny little steps, and then it doesn’t feel so incredibly overwhelming. I think that was helpful for me.”	5 (19)
Activity scheduling	“I did probably like module three the most, with this specific activity examples. I probably am somebody who spends most of my time under-aroused, except for every once in a while, I can feel that the hyperarousal too, so the skill set around scheduling activity, but then also the examples of what activity it could include, I thought that was unique and beneficial.”	5 (19)

#### Open-Ended Questionnaire Data

Table 6 provides the open-ended survey results on what clients liked or found helpful about the course and case stories. Table 7 provides open-ended survey results on what areas of the course and case stories clients wanted changed or improved. Feedback was provided by more clients on areas for improvement, potentially because areas for improvement were assessed in both the course use questionnaire and TSQ, whereas likes or helpful aspects of the course were only assessed during the TSQ.

**Table 6:** Positive feedback on *SSO^a^ Wellbeing Course* and case stories based on qualitative survey data (n=39).

Domain and category			Example quote		Clients, n (%)
**Course likes**					
	Stories, examples, and tailored to SSOs	“I found the stories most helpful in feeling understood which helped me keep on track with implementing the lesson information.”		19 (49)	
	Psychoeducation or skills	“I like the evidence-based lessons.”		14 (36)	
	Additional resources	“The handouts were also very helpful and I have printed several of them off to share with my partner.”		10 (26)	
	Helpful course or enjoyed the course	“I think that this is an amazing tool for families, and intend on using it as long as I have access to it.”		10 (26)	
	DIY^b^ guides	“DIY guides, good summary for the tools presented in each lesson.”		9 (23)	
	Convenience, accessibility, flexibility, self-guided, and no cost	“Accessible without the increased anxiety of sitting in front of someone expressing these internal thoughts and feelings. I feel the structure provided me with more of an opportunity to navigate and learn to self-reflect and self-manage my mental health concerns as they arise.”		8 (21)	
	Format	“I liked the formatting and ease of use.”		6 (15)	
**Case story likes**					
	Authentic, relatable, and realistic	“I found the stories pretty relatable as a [PSP] spouse.”		27 (69)	
	Provided comfort	“It is nice to see that you are not alone, hearing those stories are very helpful.”		9 (23)	
	Variety and diversity	“The stories are great. I appreciate the diversity represented and the various circumstances and jobs also represented.”		9 (23)	
	Helped make connections to materials or apply skills and information	“They were extremely valuable to help me put theory into practice and to help with understanding of the lesson.”		6 (15)	

^a^SSO: spouse and significant other.

^b^DIY: do-it-yourself.

**Table 7:** Constructive feedback on *SSO^a^ Wellbeing Course* and case stories based on qualitative survey data (n=81).

Domain and category		Description	Example quote	Clients, n (%)
**Overall course areas for improvement**				
	Delivery changes or additions	Inclusion of audio, video, or interactive features; additional reminder emails; personal tailoring of content delivery; putting slides into PDF format; and include workbook	“I wished I could listen to the slides or read—this way I could turn it on and listen not just have to read through them.”	15 (19)
	Content changes	Suggestions for new content to add or changes to the current content (eg, reduce length and repetitiveness); inclusion of new topics (eg, assertiveness and communication with spouse, resource on affairs, how to support spouse, and more examples); and reduce amount of information	“I would recommend a resource on affairs and recovering from affairs, as these seem to be common within the profession.”	13 (16)
	Survey issues	Suggestions to change the timing of surveys or survey response options	“Just the scheduling of these check-ins as they sometimes don’t line up with where I am at in the course, if there is a way to prompt them after I have completed a certain module that would be great.”	12 (15)
	Technical issues	Fixing issues related to being locked out of lessons, requests for slides to be continuously available, and fix issues with malfunctioning slides	“Sometimes I found the slides to be glitchy with pushing me forward.”	7 (9)
	Timelines	Requests for additional time, structure felt forced, and requests to access course for a longer period	“An occasional reprieve or extra week would reduce the trying-to-keep-all-the-balls-in-the-air guilt.”	4 (5)
	Need for more accountability or therapist support	Requests for support to work through course, need for due dates, and difficulties with self-guided format	“I’ve realized I don’t do well with completely self-guided. I need a due date otherwise I keep pushing the work back.”	3 (4)
**Case stories areas for improvement**				
	Changes to content or additional topics	Use real stories, not fictional; add more stories; include more backstories; increase variety; too much diversity; and requests to add specific topics, including supporting spouse with mental health challenges, loneliness of SSO, and more relationship stages (eg, retirement)	“I would have read more stories if they were available.”	25 (31)
	Delivery of stories	Add audio or video, remove pictures, reduce length, less abrupt endings, and systematize or ability to filter out nonapplicable stories	“Having videos of the people telling their stories would be helpful.”	17 (21)
	Relatability	Did not find the stories relatable or only found some stories relatable and stories did not provide validation	“That I felt like I didn’t relate to any. They were bigger than my issues which feels smaller but also more generic. I can’t pinpoint my struggles as easily?”	13 (16)

^a^SSO: spouse and significant other.

### Using Data to Inform Program Improvement

Areas for improvement were identified based on qualitative data, quantitative data, or both. Many changes were made based directly on qualitative client feedback. For instance, new content and revisions made to both the additional resources and case stories were informed by client feedback. Some changes were implemented specifically based on quantitative data. For example, a certificate of completion was added to increase motivation for completion, based on findings that most clients did not complete the full course. Other changes were made based on a combination of both qualitative and quantitative data. For instance, the discussion forum was removed based on interview feedback that it was not being used and quantitative data showing low use. Table 8 outlines the areas for improvement and details regarding changes made within each area.

Changes were primarily made based on the teams’ capacity to make the changes. We were unable to implement some suggestions. For instance, the request to have therapist support added to the course could not be accommodated because the course was designed as a self-guided program, and the addition of therapist support is beyond available funding. As another example, changes to the wording of clinical symptom surveys (eg, PHQ-9 and GAD-7) could not be adjusted because such an adjustment could impact the validity of these measures.

**Table 8:** Changes made to the *SSO^a^ Wellbeing Course*.

Area for improvement			Status
**Course design and materials**			
	Change the format of additional resources	Complete	
	Minor process fixes (eg, adjustments to automated emails)	Complete	
	Add the certificate of completion	Complete	
**Improve stories**			
	Add a new story character	Complete	
	Adapt the existing case stories and change character photos	Complete	
	Change the display of case stories	Complete	
**Include more topics**			
	Add new additional resources including self-care, culture and mental health, improving the couple relationship, moral injury, and supporting a partner with mental health concerns	Complete	
	Add links to PSPNET Families website to provide additional information on specific topics	Complete	
**Flexible timelines**			
	Increase to 6-month timeline	Complete	
	Adjust the release of lessons to allow for immediate access to the next lesson following completion of the previous lesson	Complete	
**Audio and video content**			
	Add audio to each lesson slide	In progress	
	Add more video content	Under discussion	
**Forum**			
	Remove forum because of low use	Complete	
**Screening and evaluation process**			
	Reduce the length of screening and evaluation questionnaires	Complete	
	Automate immediate access to the course following informed consent and screening measures	Complete	
**French translation**			
	Offer French version of the course	Complete	

^a^SSO: spouse and significant other.

## Discussion

### Summary of the Nature and Purpose of This Study

There is a growing body of research demonstrating the impacts of the occupational demands of public safety work on SSOs [4,9,10,50], highlighting a need for resources that support SSOs’ mental health and well-being [11]. In addition to providing care to a population in need of tailored supports, enhancing the well-being of SSOs may positively impact the well-being of PSP and PSP families more broadly [5]. To address the gap in services available to SSOs, PSPNET Families tailored a transdiagnostic, self-guided ICBT course for SSOs (the *SSO Wellbeing Course*). There is extensive research supporting the use of ICBT in the general population [14,15] and, more recently, for PSP specifically [35,36]. This study was a formative evaluation of the *SSO Wellbeing Course*, which represents the first specialized ICBT program created to support SSOs across Canada. This research was designed to assess the initial use and perceptions of the *SSO Wellbeing Course*. The objectives were to gain an understanding of who was enrolling in and using the course, what clients liked about the course, what components they found helpful or challenging, and their feedback regarding areas for improvement. The overall goal was to use this information to enhance the program and continue to tailor the course to the specific needs of SSOs while raising awareness of this population’s needs and informing other groups who may be developing services or resources for SSOs.

### Principal Findings and Implications

The development and continuous adaptation of the *SSO Wellbeing Cours*e was a collaborative effort between PSPNET Families clinicians and researchers, the WG, and clients of the course. In presentations regarding initial research findings and conversations with SSOs, PSP, and public safety organizations across Canada, there has been widespread interest in the *SSO Wellbeing Course*. Given the extensive positive feedback from clients, the findings of this study support the value of co-design, user-centered design principles [40], and making ongoing adaptations [51]. This study also demonstrates how mixed methods research can be used to make rapid and ongoing improvements to an intervention. The results of this study support the value of qualitative research for assessing clients’ perceptions of ICBT; for understanding the needs of a specific group; and for making ongoing, iterative improvements [35,52,53]. The additional inclusion of quantitative data alongside qualitative feedback to inform course improvements adds to the existing literature by showing how a mixed methods approach can provide further insights for course adaptations. Overall, the results reveal that many SSOs had positive perceptions of the course and found many aspects of the course helpful, indicating that the course was appropriately tailored to meet the needs of this group. The helpful aspects of the course and areas for improvement that were identified are consistent with previous qualitative research on the *PSP Wellbeing Course* [35] as well as qualitative research on the version of the *Wellbeing Course* offered to the general public in the province of Saskatchewan [53]. Similar to previous research on ICBT, the findings of this study also reflect that ICBT is not a panacea that will meet the needs of all clients [54]. Some clients felt that the course was not meeting their specific needs, and alternative intervention approaches and formats may be better suited to some SSOs seeking treatment.

Clients endorsed diverse mental health challenges, both in terms of areas of concern and severity of concerns, which supported the use of a transdiagnostic program. Previous findings from ICBT clinics demonstrate that many clients experience comorbid symptoms; therefore, the use of transdiagnostic ICBT is often preferable over a disorder-specific approach for reaching diverse populations [54]. Transdiagnostic ICBT has been shown to be as effective as disorder-specific ICBT [13,30]. Approximately half of the SSOs who enrolled in the course did not endorse clinically elevated symptoms of depression, anxiety, or PTSD, suggesting that there may have been an interest in the course for preventive or other reasons, such as supporting the mental health of their SSO working in the public safety sector. Previous research has demonstrated that ICBT can help prevent mental health concerns, such as anxiety and depression [55,56], supporting the extension of this course to SSOs who are seeking a preventive intervention.

SSOs who enrolled in the course resided in many provinces and territories across Canada and reported that their SSOs worked in various PSP sectors. This study’s sample identified primarily as White women whose SSOs worked in policing. A likely reason our sample included so many SSOs of police officers is that the number of police officers in Canada is more than double the number of PSP in most other PSP occupations, including border services officers, public safety communicators, correctional workers, career firefighters, and paramedics [57]. The interview sample generally reflected the demographics of the entire sample. Therefore, feedback on the course came from a relatively homogeneous sample, and reach to other groups is needed. Further outreach is ongoing and is required to recruit a more diverse sample. Over the course of creating PSPNET Families, we have learned that outreach and recruitment are challenging with this population. SSOs do not often identify themselves as a unique population and often see themselves as the ones who *give* rather than *receive* support, which were common themes that arose in WG discussions. Moreover, for many PSP sectors, there is no national organizational body; this introduces challenges in advertising and promoting the course to SSOs connected with certain PSP occupations. Continued engagement with relevant parties to assist in spreading the word about the *SSO Wellbeing Course* and using creative ways to reach SSOs across Canada (eg, through social media platforms and at PSP organization family events) are necessary to increase reach. Some sector-specific national organizations are only able to promote programs that are offered in both English and French. The recent completion of a French version of the course is intended to remove this barrier and to increase reach to French-speaking Canadians. The research component of the course (eg, completing questionnaires) could also hamper participation. Another potential barrier to client recruitment was that the *SSO Wellbeing Course* was offered through the PSPNET Families website, which contains extensive content and skills that are offered without registration or screening; access through the website may be hindering signup for this specific course because potential clients have immediate access to the website content. Accordingly, our team has recently relaxed the eligibility criteria for the *SSO Wellbeing Course* and streamlined the eligibility screening process (eg, reducing the number of questionnaires) to facilitate quicker access to the course for new clients. Additional pathways for course signup on the PSPNET Families website as well as ways to connect website users to the service that best suits their needs are under consideration.

Both quantitative and qualitative results suggested that relationship dissatisfaction was a common concern among SSOs seeking ICBT. On the basis of this information, additional resource content was created to address both general and specific (eg, intimacy issues, loneliness, and infidelity) relationship concerns. The PSPNET Families team continues to monitor whether the addition of this information meets the needs of course clients or whether additional changes are required (eg, adding a separate lesson on relationship challenges). Given that relationship concerns were a primary challenge reported by SSOs, other services designed to support SSOs may benefit from explicitly assessing and addressing relationship challenges within this population.

All 16 additional resources were viewed by multiple clients, suggesting that the additional resource topics were relevant to this population. The additional resource with the most unique viewers was on communication with an SSO. On the basis of client feedback, additional content was created to address the topics of supporting a partner with mental health concerns and specific relationship challenges (ie, intimacy issues, loneliness, and infidelity). Additional content on retirement was suggested but was determined to be outside the scope of this project. The PSPNET Families team is considering ways to address this topic (eg, adding content to the PSPNET Families website) to meet the needs of SSOs in this career stage.

One component of the course that had little uptake was the discussion forum. A therapist-moderated discussion forum was included as an optional component of the course to facilitate peer support and potentially improve outcomes, based on previous studies showing that unguided ICBT offered with a web-based discussion forum shows equivalent outcomes to guided ICBT [58-60] and previous research suggesting that forums may be particularly beneficial for SSOs [5]. However, the forum was ultimately removed from the course because of low use and client feedback. Other ways to potentially increase forum engagement (eg, making it more easily accessible on the PSPNET Families external website) are under consideration, and depending on the security of longer-term funding, the forum may be reinstated in the future.

With respect to barriers to completing the course, a few SSOs (4/81, 5%) indicated that time constraints were a primary challenge. Similar challenges were experienced by PSP in the *PSP Wellbeing Course* [35]. On the basis of client feedback, there was a need to increase flexibility with course timelines to accommodate SSOs, which is consistent with previous research indicating that time-based stressors are often experienced by SSOs because of the scheduling demands of PSP work [11]. These insights can help inform other service providers about potential barriers regarding internet-delivered supports for SSOs and the importance of flexible timelines when providing support to PSP families. To address timeline challenges in the *SSO Wellbeing Course*, the release of lessons was adjusted to allow for immediate access to the next lessons following the completion of a previous lesson (instead of a gradual release of lessons over 8 weeks). An extension to the course timeline (from 8 weeks to 6 months after enrollment) was also implemented. We did not eliminate timelines entirely because previous research indicates that some timelines are needed to ensure accountability with course completion and to improve outcomes [61]. Of the 118 clients who started the course, 71 (60.2%) clients completed the majority of the lessons. To encourage course completion, a certificate of completion was added.

ICBT addresses many barriers associated with face-to-face therapy [18,19]. ICBT offers treatment in a convenient and accessible format, which can be particularly advantageous for SSOs who live in rural or remote areas with limited access to specialized services. Most clients (72/116, 62.1%) reported living in rural or small communities across Canada. In the interviews and open-ended surveys, *convenience* and *accessibility* were frequently cited as reasons why ICBT worked for SSOs or as helpful aspects of ICBT. These findings support the idea that more services are needed for SSOs [11], particularly those that are accessible and can address the unique needs of SSOs (eg, address geographical and time constraints, are developed using knowledge about PSP families’ unique lifestyles, and confidentiality concerns).

### Limitations and Future Research

There are a number of limitations of this study that require consideration. First, the sample primarily comprised SSOs who identified as White women. Future research should focus on collecting data from SSOs with diverse backgrounds to increase the generalizability of the findings and to enhance the program by ensuring that the course captures diverse experiences and meets the needs of SSOs from across Canada. This can be achieved through increased promotion of the course to SSOs with diverse backgrounds and through purposive sampling. Second, although the clients who participated in the interviews appeared representative of the total sample in most respects, their rate of course completion was nearly double that of the total sample. It is possible that clients who were less engaged with the course were less likely to agree to an interview, and it is also possible that clients made an effort to engage with the course more after agreeing to an interview; in either case, there was minimal representation of low-engagement clients among the interviewees. Third, although most of those who participated in the interviews completed the course (ie, all 5 lessons), the interviews took place at various stages of course completion. Therefore, answers to questions querying course helpfulness and areas for improvement would be limited for SSOs who had not yet completed the course, which was beneficial in capturing perspectives on barriers to course completion. Fourth, this study focused on a formative evaluation of the *SSO Wellbeing Course*. Although this is an important first step to assessing and improving the course, future research should be conducted to investigate both short-term and long-term clinical outcomes (eg, symptom changes) and potential impacts on the relationship (eg, changes in relationship satisfaction and function) and the broader PSP family. In addition, a few clients requested the addition of therapist support to the course, a change that could not be accommodated in this study. Future research could expand to include therapist-assisted ICBT programs for SSOs and compare use, perceptions, and outcomes between self-guided and therapist-guided ICBT for SSOs.

### Conclusions

To our knowledge, this formative evaluation of the *SSO Wellbeing Course* is the first study of a self-guided, transdiagnostic ICBT program tailored to SSOs of PSP. The results indicated that most clients had positive feedback about the course, including finding the course and specific skills helpful, liking the case stories, and appreciating the convenience and accessibility of ICBT. Clients also provided feedback on areas for improvement, which largely represent common themes related to how ICBT can be improved (eg, more audio-visual content and improved timelines). Some unique suggestions, however, were identified related to some content (eg, how to support a spouse with mental health needs, more information on moral injury, and ensuring that content is appropriate for those taking the course for preventive as well as clinical reasons). Both client feedback and quantitative data were used to make iterative changes to enhance the course. Most clients identified as White women, suggesting a need to reach more diverse groups of SSOs. This study highlights the importance of tailoring support services to the experiences of SSOs and provides knowledge about the unique needs of this population that can help inform other services designed to support SSOs and PSP families.

## Abbreviations

ICBT: internet-delivered cognitive behavioral therapy
GAD-7: Generalized Anxiety Disorder-7
PHQ-9: Patient Health Questionnaire-9
PSP: public safety personnel
PTSD: posttraumatic stress disorder
SSO: spouse and significant other
TSQ: treatment satisfaction questionnaire
WG: working group

## Data Availability

Clients were informed, before consenting to participate in this research, that their data would be stored on a secure server and would not be shared with other parties outside of PSPNET. Therefore, we are unable to share data.
